# Evaluation of the recombinant antigens Wb14 and WbT for the capture antibody diagnosis of lymphatic filariasis

**DOI:** 10.1590/0074-02760170435

**Published:** 2018-03-26

**Authors:** André Filipe Pastor, Abraham Rocha, Klécia de Melo Cassemiro, Marli Tenório, Paula Melo, Maria Rosângela Grilis, Maressa Rhuama, Antonio Mauro Rezende, Osvaldo Pompilio de Melo, Ernesto Marques, Rafael Dhalia

**Affiliations:** 1 Fundação Oswaldo Cruz-Fiocruz, Instituto Aggeu Magalhães, Recife, PE, Brasil; 2 Universidade Federal de Pernambuco, Recife, PE, Brasil; 3 Fundação Oswaldo Cruz-Fiocruz, Instituto Aggeu Magalhães, Serviço de Referêcia Nacional em Filarioses, Recife, PE, Brasil; 4 Instituto Federal de Ciência e Tecnologia do Sertão Pernambucano, Campus Floresta, PE, Brasil; 5 Hospital Otávio de Freitas, Recife, PE, Brasil; 6 Instituto Nacional de Câncer, Labratório de Patologia Clínica, Rio de Janeiro, RJ, Brasil

**Keywords:** immunodiagnostic, Wuchereria bancrofti, ELISA, biotechnology

## Abstract

**BACKGROUND:**

Lymphatic filariasis (LF) is a parasitic disease caused mainly by the *Wuchereria bancrofti* worm and that affects up to 120 million people worldwide. LF is the second cause of chronic global deformity, responsible for 15 million people with lymphedema (elephantiasis) and 25 million men with scrotal hydrocele. Its diagnosis is still associated with numerous difficulties, such as the sample collection periods (microfilaria nocturnal periodicity) and limited diagnostic kits.

**OBJECTIVES:**

The aim of this work was to evaluate two recombinant antigens (Wb14 and WbT) as part of an enzyme-linked immunosorbent assay (ELISA) based antibody capture tests for LF.

**METHODS:**

The recombinant antigens rWb14 and rWbT were expressed in *Escherichia coli* BL21 and an antibody capture ELISA was performed. For this, sera were used from microfilaremic individuals with *W. bancrofti* (MF), chronic pathology (CP), individuals infected with *Strongyloides* (SP) and healthy controls from endemic (EN) and non-endemic (NE) areas.

**FINDINGS:**

Both tests showed similar results, with 90% sensitivity and 96.6% specificity. In comparison with the BM14 ELISA commercial test, the Wb14 and WbT antigens performed with identical sensitivity but greater specificity. Reduced positivity with the CP suggested a potential to monitor cure. This was not confirmed, however, when sera from individuals up to seven years after treatment were assayed.

**MAIN CONCLUSIONS:**

The Wb14 and WbT ELISAs were considered efficient and promising diagnostic tests. Due to the importance of antibody capture analysis to evaluate the Global Program to Eliminate Lymphatic Filariasis (GPELF), the tests proposed here appear as great alternatives to the available commercial system.

Lymphatic filariasis (LF) is a parasitic disease caused by nematode worms belonging to the genera *Wuchereria* or *Brugia* and transmitted by a great number of mosquito species (WHO 2016). It affects over 100 million people worldwide and 90% of the reported cases are attributed to *W. bancrofti*. LF is the second cause of chronic global deformity, responsible for 15 million people with lymphedema (elephantiasis) and 25 million men with scrotal hydrocele (WHO 2010).

In 1993, the World Health Organization (WHO) listed LF as one of six potentially eradicable infectious diseases. Later, in 1997, the World Health Assembly (resolution 50.29) elected it as a potentially eradicable global public health problem. In response to this resolution, WHO launched in 2000 the Global Program for Elimination of Lymphatic Filariasis (GPELF), which aims to eliminate the disease until 2020 (WHO 2010). To evaluate the success of GPELF, it is essential to monitor the breakdown of LF transmission through diagnostic methods able to detect the disease with high sensitivity and specificity. These should be capable of rapidly identifying residual foci of infection that can be blocked to prevent the disease resurgence. WHO has thus implemented guidelines and protocols to map, monitor and evaluate the success of GPELF through appropriate laboratory tests (Ottesen 2000).

In 2000, when GPELF was created, the diagnostic methods for LF were limited to clinical examination, detection of antibodies against crude antigen preparations and visual detection of microfilaria from capillary and venous blood samples, the latter using the techniques of thick smear and membrane filtration, respectively. The thick smear approach has been used worldwide for several decades because it is a low cost technique that demands little infrastructure (Ramzy 2002). However, in areas where *W. bancrofti* is endemic and the periodicity of the microfilaria is nocturnal, this diagnostic approach faces difficulties due to the need for blood collection at late hours. A resistance by the targeted communities to blood collection might thus occur due to religious beliefs, violence or the inconvenience of the late night approach. In addition, this test may be unable to confirm infection in individuals with low microfilaria density or even temporarily amicrofilaremic, but nevertheless with the potential to contribute to future transmissions (Ximenes et al. 2014). An alternative immunodiagnostic method developed almost thirty years ago for LF diagnosis was the Og4C3 ELISA, based on the search for circulating filarial antigens (More and Copeman 1990). More recently, the point of care immunochromatographic AD12 card test was developed (POC-ICT) (Weil and Lammie 1997) and it has now been replaced by the Filariasis Test Strip (FTS) (WHO 2016). These methods have a greater sensitivity for circulating antigens detection and blood samples can be collected at any time of the day (Weil and Lammie 1997, Rocha et al. 2009). However, individuals can remain positive for many months after cure and the tests may still give a false negative result for samples with low microfilaria densities (Iqbal and Sher 2006). In addition, both microfilaria and circulating antigens appear only several months after infection, limiting the use of these tests to monitor the decrease in transmission intensity or even resurgence of LF in later stages after elimination (Damgaard et al. 2016).

To overcome the limitations of microfilaria and antigen capture tests, the screening for anti-filarial antibodies can be used as a marker of residual endemicity or the onset of a resurgence of transmission, functioning as a warning system in areas that have been subjected to LF eradication measures (Joseph et al. 2011). Tests have been developed based on the use of recombinant antigens to detect anti-filarial antibodies. Among those antigens, two of the best known are from *W. bancrofti*, WbSXP-1 (Rao et al. 2000) and Wb123 (Kubofcik et al. 2012) and one from *B. malayi*, Bm14 (Lammie et al. 2004). The latter gave rise to the most widely used antibody capture kit for LF diagnosis, the ELISA BM14 (CELISA) (Weil and Ramzy 2007, Weil et al. 2011, Nunes et al. 2016). However, cross-reactivity issues have been reported with the ELISA BM14 test (Weil et al. 2011) and the fact that the recombinant antigen is derived from *B. malawi*, instead of *W. bancrofti*, may lead to some false negative results. The present study aimed to solve these issues by evaluating two further recombinant antigens derived from *W. bancrofti*, Wb14 and WbT, against a panel of sera from patients afflicted with LF and controls and considering their use in an alternative antibody capture test for the diagnosis of the disease. Such test would be extremely useful to evaluate the success or failure of the GPELF in several endemic areas where control interventions have been taking place, especially considering that the lack, until recently, of efficient alternatives to the ELISA BM14 test have led to difficulties in its acquisition and use for large scale LF diagnosis, in Brazil at least.

## MATERIALS AND METHODS

*Ethical aspects* - This study was approved by the Research Ethics Committee from the Institute Aggeu Magalhães, FIOCRUZ-PE (CEP - 45085215.0.0000.5190).

*Serum bank* - Aliquots from the 114 sera evaluated here were obtained from a bank of LF biological samples stored at -20ºC and belonging to the Brazilian National Filariasis Referral Service, based at the Institute Aggeu Magalhães (FIOCRUZ-PE) (Rocha et al. 2009). The participants (or their parents in the case of minors) were given information about the research and were asked to read and sign the terms of consent.

*Laboratorial assays for filarial investigation* - Briefly, 10 mL of venous blood were first split into two ~5 mL aliquots in the presence of EDTA. The first aliquot was used for the visualisation and quantification of microfilaria after filtration while the serum from the second aliquot was used for serological assays. For the ELISA Og4C3 (TropBio®, JCU Tropical Biotechnology Pty Ltd, Townsville, Queensland, Australia) and POC-ICT (BinaxNOW, Binax, Inc., Maine, USA) tests, 100 µL of serum was placed in the position recommended for the test and a trained technician read the result precisely 10 min after loading. The visualisation of the two lines (test and control) was interpreted as a positive result. For the anti-filarial Bm-14 (Filariasis CELISA, Cellabs Pty. LTd., Brookevale, Australia tests) test and the two ELISA based assays, anti-Wb14 and anti-WbT (SRNF-Protocol), all the procedures were developed according to the manufacturers’ protocols, described elsewhere (More and Copeman 1990, Weil and Lammie 1997). Positive sera were defined as sera positive for all other previous tests evaluated (Bm14, Og4C3, POC-ICT and membrane filtration), while negative sera were also those having negative results for the same tests.

*Design, chemical synthesis, cloning and expression of the Wb14 and WbT genes* - The amino acid sequence of the Wb14 protein found in the Broad Institute database was back-translated with the help of the Gene Designer 2.0 program. WbT is derived from the same Wb14 gene but removing the nucleotides encoding the 17 amino acids subsequent to the first methionine. Restriction sites for the KpnI and NheI enzymes were then added flanking the protein coding regions for both Wb14 and WbT antigens and the final nucleotide sequences ordered from GeneArt Gene Synthesis (Invitrogen). The final gene products were then cloned into the KpnI/NheI sites from the pRSET-A expression vector (Invitrogen), a plasmid that adds a six histidine tag to N-terminus of the recombinant protein. Protein expression and purification were performed as described below. Briefly, to perform the large-scale induction of the rWb14 and rWbT proteins, 200 mL of *Escherichia coli* BL21(DE3) cells transformed with the recombinant plasmids were grown in LB medium plus 50 mg/mL ampicillin up to an absorbance of 0.8. IPTG (1 mM) was then added to the medium and growth was maintained for four more hours at 25ºC. The cells were then harvested by centrifugation and resuspended in 20 mL of PBS, followed by lysis through sonication. The purification of rWb14 and rWbT was done by immobilised metal ion affinity chromatography (IMAC) using nickel agarose beads (from Qiagen), capable of capturing the heterologous proteins due to their affinity to the proteins’ histidine tail, as recommended by the manufacturer. For confirmation of the recombinant proteins’ expression, SDS-PAGE polyacrylamide gel electrophoresis was performed. A yield of roughly 500 µg of protein per batch of purification was obtained.

*Antibody capture tests* - These were generally done as previously described for similar antibody capture assays (Rao et al. 2000, Weil et al. 2011, Kubofcik et al. 2012) with some modifications. Briefly, a 96 wells microplate (Corning 3690, Costar, USA) was sensitised with 50 μL of the recombinant antigen at a concentration of 20 μg/mL in 0.1M Carbonate /Bicarbonate buffer (pH 9.6) and incubated for 18 h at 4ºC. Blocking was then performed with phosphate-buffered saline (PBS - pH 7.2) supplemented with 4% BSA, followed by five washes with PBS plus 0.05% Tween 20 (PBS / T). For the assays, 100 μL of serum diluted 1: 100 in PBS supplemented with 0.5% BSA were loaded per well, followed by incubation at 37ºC for 1 h. After a new set of washes 50 μL of HRP Mouse Anti- Human IgG4 (Zymed) diluted 1: 15,000 in PBS plus BSA was added to each well followed by another 1 h incubation. Following a final set of washes, the BD OptEIATM TMB substrate (BD Biosciences, USA) was added and the plates incubated for 15 min at room temperature, with the reaction stopped with 50 μL of a 1N H_2_SO_4_ solution. The plates were read at 450 nm using the Benchmark Plus microplate spectrophotometer (BIORAD, USA) and the results were expressed in optical density (OD).

*Statistical analysis* - All graphs were made using the GraphPad prism software version 6.0 (GraphPad Software, San Diego, CA, USA). The mean absorbance values for each individual were used to calculate the IgG4 antibody response against Wb14 and WbT. The cutoff was determined as the mean absorbance value derived from the assays using the sera from the true negative (NE) group plus three fold the standard deviation. The sensitivity, specificity and the positive and negative predictive values were determined using the results from the true positive and true negative groups. In addition, a receiver-operating characteristic (ROC) curve was made to determine the accuracy of each test.

## RESULTS

**Fig. 1:**
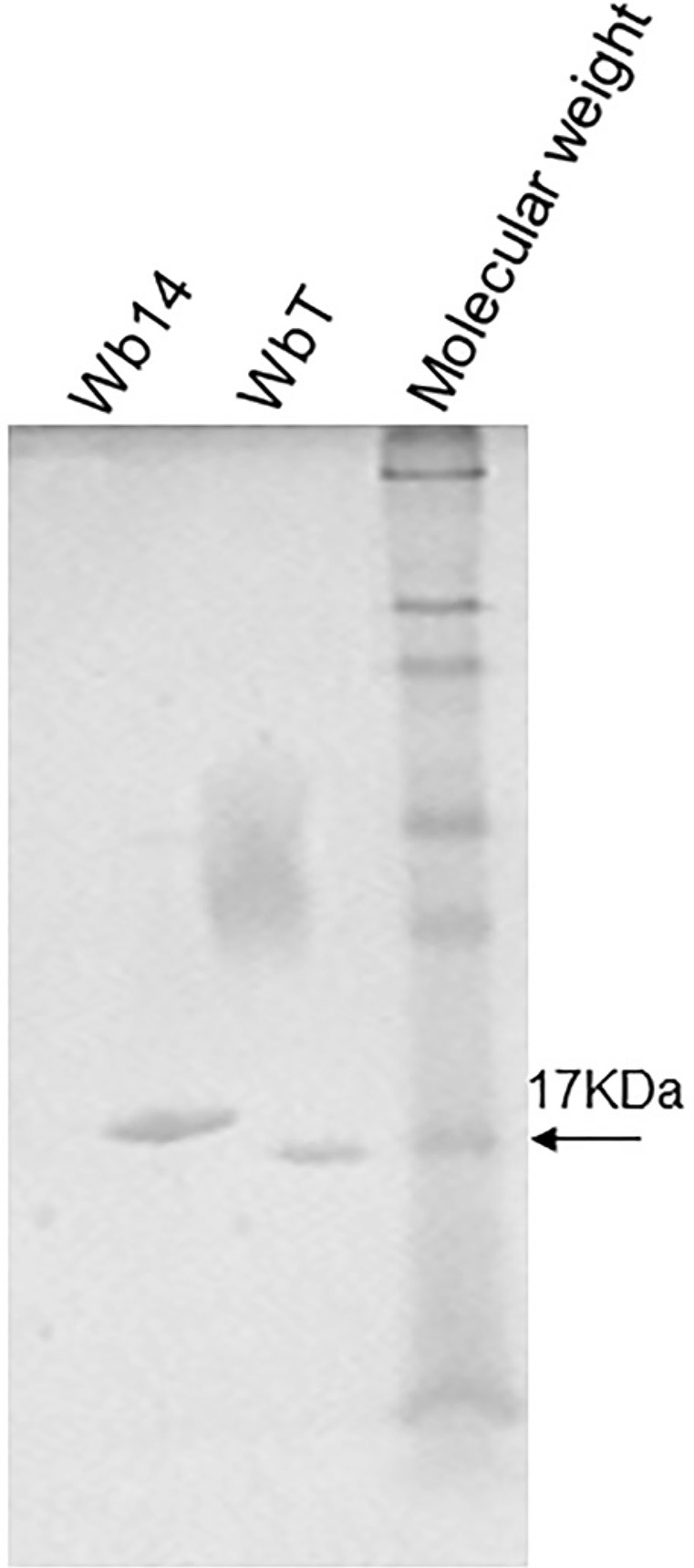
: polyacrilamide gel electrophoresis evaluating the affinity purified Wb14 and WbT recombinant polypeptides. His-tagged, recombinant Wb14 and WbT were ran on standard 12% SDS-PAGE gels stained with Coomassie Blue. Prestained molecular weight markers are shown on the right (7-175 kDa) with the 17 kDa band indicated by the arrow. The smear seen in the WbT lane is an artifact introduced inadvertently during the figure preparation.

*Expression of recombinant Wb14 and WbT* - In the present work, the recombinant Wb14 antigen from *W. bancrofti* was expressed in *E. coli* with an N-terminal poly-histidine tag. At the same time, an artificially truncated form was created through the removal of the 17 residues localised immediately after its first methionine, generating a different recombinant antigen, the WbT. This was done because this region proved to be very hydrophobic, which could prevent an efficient expression of the antigen as well as its recognition by the human sera. After large scale induction and affinity chromatography the two recombinant polypeptides were ran on SDS-PAGE gels to monitor their integrity (Fig. 1). As expected according to their predicted sizes, the Wb14 band ran with an apparent molecular weight slightly larger than 17 kDa (~18 kDa) while the WbT polypeptides migrated with a size very similar to the 17 kDa molecular weight marker. The size observed for the recombinant Wb14 is smaller than the one observed for the previous report with the same protein (Pandiaraja et al. 2010a), however this may be due to differences in the subcloning strategy or even SDS-PAGE methods. No indication of degradation is seen for either protein.

*Evaluation of Wb14 and WbT antigens for the diagnosis of FL* - To evaluate the potential use of the antigens produced here for the FL diagnosis, sera from different groups of individuals were evaluated for their ability to recognise both polypeptides through enzyme-linked immunosorbent assay (ELISA). Five different groups were selected according to previous clinical and parasitological evaluations and classified into distinct profiles: microfilaremic individuals (MF; n = 30); chronic pathology (CP; n = 26); individuals positive for *Strongyloides* sp. and other intestinal parasites (SP; n = 13); healthy individuals from the endemic region (EN; n = 15); healthy controls from non-endemic regions (NE; n = 30). For these assays the microfilaremic group was considered true positive and the non-endemic group as true negative.

**Fig. 2:**
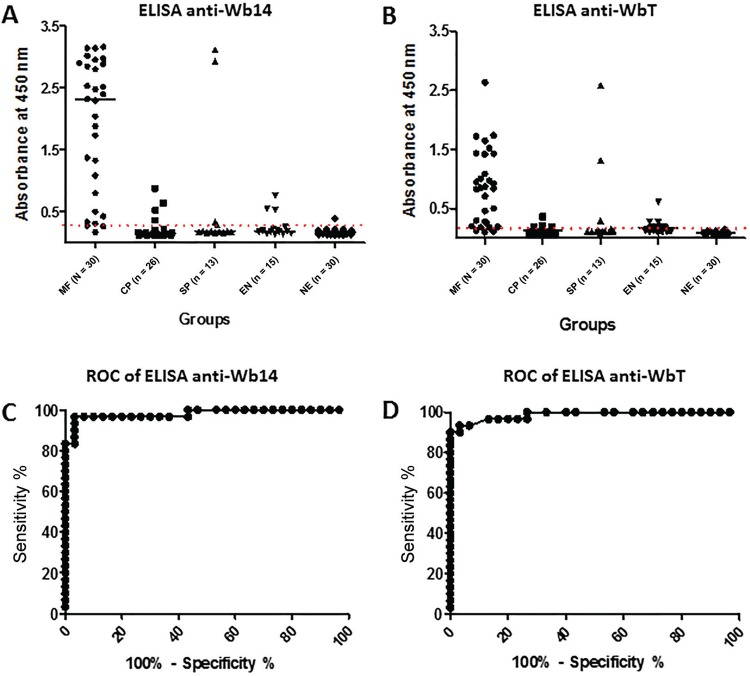
: enzyme-linked immunosorbent sssay (ELISA) evaluating the recognition of the two recombinant proteins by different groups of lymphatic filariasis (LF) related sera. (A, B) Summary of the ELISA results for the Wb14 and WbT polypeptides, respectively. Each point represents a different serum within the various groups evaluated: MF: micofilaremic individuals; CP: chronic patients; SP: *Strongyloides* positives; EN: endemic normal; NE: non endemic. The cutoff is represented by dashed lines: 0.37 for Wb14 ELISA and 0.14 for the WbT ELISA. (C) receiver-operating characteristic (ROC) curve evaluating the performance of the Wb14 and WbT antigens. The area under the curve was 0.9811 (98.11% accuracy) and 0.9867 (98.67%) for the two antigens, respectively, with a standard deviation of 0.01575 and 0.01085, and a confidence interval of 0.9502 to 1.012 and 0.9654 to 1.008. The p-value was < 0.0001 for both.

The ELISA results for the two antigens with the various groups are summarised in Fig. 2A, B and Table. Wb14 is efficiently recognised by most sera from the true positive microfilaremic group with 90% of the sera (27/30) producing a positive result. In contrast, for the second group with chronic pathology only 19.2% of the sera were positive (5/26). For those sera from individuals with *Strongyloides*/intestinal parasites a similar result was observed with a 23% positive result (3/13), but two of those positive sera produced very high absorbance values, suggesting a cross-reactivity. Likewise, the first group of negative sera, from the endemic region, also had a 20% positivity result (3/15), but here the absorbance values for the positive sera were low. In contrast, for the true negative group from the non-endemic region, only 1 out of 30 sera (3.3%) was positive in the assay, even then with a very low absorbance, just above the cutoff.

**TABLE:** Summary of the data from the various tests performed in order to compare their performance for the diagnosis of lymphatic filariasis. The numbers of positive sera are shown for each group and test, as well as the sensitivity, specificity, positive predictive value (PPV) and negative predictive value (NPV) derived from the data based on the comparison between the microfilaremic (MF) and non-endemic (NE) groups. The others groups are chronic patients (CP); *Strongyloides* positives; endemic normal (EN)

Tests Groups	Wb14 ELISA	WbT ELISA	Bm14 ELISA	Og4C3	POC-ICT	Filtration
MF	27/30	27/30	27/30	28/30	27/30	30/30
CP	5/26	5/26	16/26	0/26	1/26	0/26
SP	3/13	4/13	3/13	1/13	1/13	0/13
EN	3/15	5/15	4/15	0/15	0/15	0/15
NE	1/30	1/30	9/30	0/30	0/30	0/30
Sensitivity (%)	90%	90%	90%	93.33%	90%	100%
Specificity (%)	96.6%	96.6%	70%	100%	100%	100%
PPV (%)	96.4%	96.4%	75%	100%	100%	100%
NPV (%)	90.6%	90.6%	87.5%	93.7%	90.9%	100%

The ELISA using the WbT antigen produced results very similar to those from Wb14 but in general with lower absorbance values and cutoff, compatible with the fact that WbT is a truncated form of Wb14 (Fig. 2B). Minor differences were observed for the *Strongyloides*/intestinal parasites and the healthy control group from endemic areas where a slight increase in positivity was observed for WbT (also summarised in Table). For those groups, however, the same sera that produced the highest absorbance values with the Wb14 antigens were also the same that led to higher absorbance with WbT. Overall performance by both antigens was then evaluated through a ROC curve analysis. For the Wb14 ELISA, it revealed a 98.11% accuracy for the test (p < 0.0001), with a very good performance (Fig. 2C). For the WbT ELISA its performance was even better with a 98.67% (p < 0.0001) accuracy result (Fig. 2D).

**Fig. 3:**
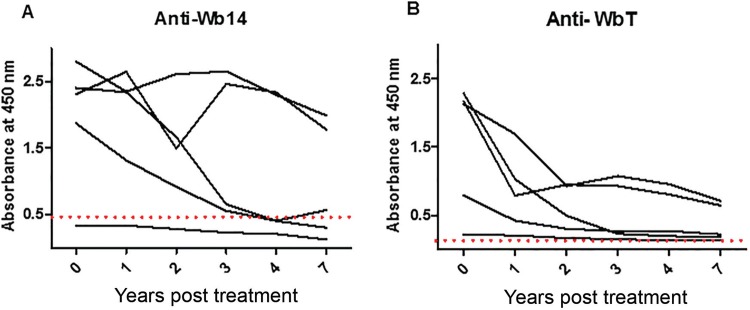
: evaluation of the antibody titer against the Wb14 and WbT recombinant antigens at different time periods after diethylcarbamazine (DEC) treatment. The same five individuals were tested for both antigens before treatment with DEC and at several time points thereafter (1, 2, 3, 4 and 7 years). The cutoff is represented by the dashed line.

*Monitoring anti-Wb14 and anti-WbT antibodies after DEC treatment* - The “chronic pathology” group evaluated in the Fig. 2 presumably includes individuals who were exposed to the filarial parasite, might have been infected, but were treated and/or cured of the disease a substantial amount of time before the serum samples were collected. The differences in positivity observed when the results from the microfilaremic individuals (90%), with active filariasis, are compared with those from this group (19.2%) then might indicate that the ELISA using either Wb14 and WbT could be considered as a test to monitor cure of the disease. To evaluate this possibility, the availability of serial serum samples from the same microfilaremic individual taken at specific periods after diethylcarbamazine (DEC) treatment and cure was considered. Serum samples from five randomly selected individuals collected one, two, three, four and seven years after treatment were thus evaluated with the Wb14 and WbT ELISA tests and compared with sera collected prior to treatment. The results, summarised in Fig. 3, indicate that the tests failed to establish a relationship between cure and negativity, although a clear decay in absorbance levels was observed for all five sets of sera with both antigens. For the Wb14 antigen (Fig. 3A), the absorbance values for three individuals were below the cutoff after four years of treatment, but one of these had low absorbance values, below the cutoff even in the pre-treatment sample. The other two individuals were clearly positive in all samples, despite a trend for the reduction in absorbance values with time. A similar observation was done with the WbT antigen (Fig. 3B). Despite a decay in absorbance values after seven years for all five individuals, none of them fell below the cutoff, and were all considered positive by the test. Nevertheless, a more uniform reduction in absorbance values is observed with time for the five individuals using this antigen and it is possible that, in samples older than 7 years, all five individuals would produce negative results with this test.

*Comparison of the Wb14 and WbT ELISA with tests commonly used for the diagnosis of lymphatic filariasis* - To compare the performance of the ELISA tests proposed here with current tests used for the diagnosis of lymphatic filariasis, the same sera groups assayed in Fig. 2 were also tested with four assays commonly used to diagnose the disease: BM14, the reference antibody capture test, more comparable to the assays performed here with Wb14 and WbT; Og4C3, a reference quantitative antigen capture test; POC-ICT, used as qualitative antigen capture test; and polycarbonate membrane filtration, the reference for the parasitological test. In comparison with the CELISA BM14 assay (Table), the tests described here were equivalent in sensitivity (90% for all three assays) but with a much higher specificity (96.6% for both Wb14 and WbT tests with 70% for the CELISA BM14). This was due to the fact that nine sera from the true negative group reacted with the BM14 assay while only one gave a positive result with either Wb14 or WbT. A significantly higher number of positive sera from individuals with chronic pathology were also seen for BM14 (61.5% versus 19.2% for both Wb14 and WbT). In contrast the three ELISA tests performed very similar with the other two groups assayed, with minimal differences in the number of positive samples from the patients with *Strongyloides*/intestinal parasites or the negative control individuals from the endemic area. The antigen capture tests (Og4C3 and POC-ICT) displayed sensitivities similar to the three, ELISA based, antibody capture assays, but 100% specificity when the microfilaremic group was compared with the sera from non-endemic healthy individuals, since no non-endemic serum was positive. As expected, the polycarbonate membrane filtration test produced the best results, with 100% sensitivity and specificity and no false positive or false negative results.

## DISCUSSION

Prior to the advent of molecular biology, the antibody capture tests for the diagnosis of lymphatic filariasis were based on the crude preparations of protein extracts from the worms that caused the diseases (Almond and Parkhouse 1986, Maizels et al. 1987). These tests were difficult to perform due to the laborious preparation of the extracts and their performance was affected by the high number of false positive results, due to cross reactions resulting from the presence of the many proteins and other components found in those extracts. Recombinant antigens then appeared as a promising alternative for the development of an antibody capture, ELISA based method, to diagnose LF, the most important being Bm33 (Dissanayake et al. 1993), Bm14 (Chandrashekar et al. 1994), WbSXP-1 (Rao et al. 2000) and Wb123 (Kubofcik et al. 2012). The current manuscript evaluates the Wb14 antigen, and its WbT variant, as an alternative target for ELISA based, antibody capture test to be used for the point of care diagnosis of lymphatic filariasis. Wb14 is related to WbSXP-1 and both belong to a family of nematode proteins identified as potent immunogens in many parasite infections (Rao et al. 2000) but their functions have not yet been determined.

Wb14 is a naturally truncated form of the WbSXP-1 protein from *W. bancrofti* with a stop codon at the nucleotide position 460 of its protein coding sequence. Wb14 is 153 amino acids long, while WbSXP-1 has an additional C-terminal extension of 29 amino acid residues. The two proteins share a 98% sequence similarity at the nucleotide level and both are homologous to equivalent SXP genes from related parasites, such as *B. malayi* (Bm14 and SXP-1), *Onchocerca volvulus* (Ov17) and *Ascaris suum* (As14) (Pandiaraja et al. 2010a). The results described here confirm that ELISA assays based on Wb14 and on its WbT variant perform with similar sensitivity and greater specificity for *W. bancrofti* than the well-established BM14 CELISA test (Weil and Ramzy 2007, Weil et al. 2011, Nunes et al. 2016). The Bm14 antigen was originally assayed with sera from individuals with LF from regions of India and Egypt, with 90% of the sera reactive against this antigen and cross-reactions seen only with *O. volvulus* (Chandrashekar et al. 1994). Later similar result were obtained with sera from various origins, when the sensitivity of the Bm14 ELISA was 91% for *W. bancrofti* and 96% for *B. malayi*, with cross reaction with *O. volvulus* and *Loa loa* infected sera (Lammie et al. 2004). Yet another study evaluated the CELISA Bm14 test, in which 91% of samples from *Brugia* and 98% from *W. bancrofti* were recognised, although a small cross-reaction with individuals infected with *Strongyloides* and *Ascaris* was observed, while the non-endemic group was not recognised (Weil et al. 2011). Another antigen early on proposed to be used for the diagnosis of lymphatic filariasis was Bm33, recognised by only 71% of the sera from individuals infected with *W. bancrofti* when it was first described (Dissanayake et al. 1993). A subsequent study using this antigen, however, confirmed that it could clearly discriminate between microfilaremic individuals and the non-endemic control group, although significant reactions were observed with the individuals with chronic pathology, in contrast to what was observed here (Krushna et al. 2009). More recently, the Wb123 antigen from *W. bancrofti* L3 larvae was proposed as a good choice for the analysis of newly infected persons with a subsequent rapid anti-Wb123 antibody capture test developed to identify individuals who came into contact with the *W. bancrofti* L3. These tests recognised 91.5% of the sera infected with *W. bancrofti* and showed some cross reaction with *O. volvulus* sera, but not with *L. loa* nor with subjects with *Strongyloides* (Steel et al. 2013).

Both Wb14 and WbSXP-1 have been previously evaluated for the LF diagnosis, with very similar results regarding the recognition of total human IgG in sera from microfilaremic individuals (Pandiaraja et al. 2010a). WbSXP-1 was first evaluated for its potential to diagnose lymphatic filariasis through an IgG4 antibody capture ELISA, in assays displaying a 91-100% sensitivity for sera from individuals infected with *W. bancrofti*, with a lower performance for *B. malayi*. Sera from chronic, endemic and non-endemic samples were not recognised, but cross reaction was reported with samples derived from *O. volvulus* and *L. loa* infections (Rao et al. 2000, Baskar et al. 2004, Lammie et al. 2004, Janardhan et al. 2011). Subsequently, a study was conducted that tested both WbSXP-1 and Wb14 against only five human serum samples in the ELISA, all with previously confirmed diagnosis and hydrocele. The two proteins showed similar reactivity against serum human IgG, especially from microfilaremic individuals (Pandiaraja et al. 2010a). At the same time, four synthetic peptides derived from WbSXP-1 showed significant potential as part of an antibody capture test. The one peptide with the lowest absorbance values in microfilaremic samples was located within the 29 amino acids segment that differs between Wb14 and WbXSP-1 (Pandiaraja et al. 2010b).

The present work can be justified by the absence of further followups to the previous study describing the Wb14 antigen and evaluating the human IgG response against this antigen with only five samples (Pandiaraja et al. 2010a). Here, the number of positive sera evaluated is significantly increased, to 30 microfilaremic individuals, and the results are more robust. When compared with WbSXP-1, Wb14 has the potential to be less-cross reactive since the 29 amino acids from the WbSXP-1 C-terminus missing from Wb14 has homology to equivalent sequences from many pathogens, including some species of *Plasmodium* and *Leishmania infantum* (Pandiaraja et al. 2010a). WbT is proposed as yet another new alternative for the diagnosis of lymphatic filariasis, since the three peptides that showed the best results in the previous recognition of filaremic individuals by WSXP-1 (Pandiaraja et al. 2010b) are present in the region common to WbT and the removal of the first 17 hydrophobic amino acids from Wb14 may facilitate its expression as well as recognition by the antibodies. The results shown here indicate that the anti-Wb14 and anti-WbT ELISAs may be more effective in evaluating active infections in adults. These tests also appear to be a better alternative to identify recent infections in children, since antibodies are rapidly produced in newly infected individuals (Lammie et al. 2004, Weil and Ramzy 2007, Weil et al. 2011, Hamlin et al. 2012). Regarding the permanence of positivity after treatment, there is a real difficulty in differentiating between active and past infection in antibody capture tests, since chronic patients titers remain for a long time (Damgaard et al. 2016). This has been reported with the BM14 based test, which detected as positive 16 of the 26 individuals in the chronic pathology group. Of the 16 positives, 12/14 were hydrocele carriers, while only 4/12 had lymphedema. However, in addition to the higher hydrocele ratio, these individuals who remained positive lived in regions of high-transmission for lymphatic filariasis (de Souza et al. 2015), which may allow reinfection (Terhell et al. 2003). Although few chronic individuals were recognised as positive in the Wb14 and WbT ELISAs, both tests developed here were unable to predict cure in samples of *W. bancrofti* infected after seven years of DEC treatment and these discrepancies will have to be investigated further. It is possible that, at least for the two individuals that experienced an increase in antibody after the expected decline, this may be the result of reinfection or recovery of resident adult worms following DEC treatment, as described by Terhell et al. (2003). Nevertheless, antibodies against the Wb123 antigen failed to define negativity in samples from two Guyana-infected individuals, even after 17 years of treatment and without returning to an endemic *W. bancrofti* area during that period (Kubofcik et al. 2012).

In summary, our results indicate that the anti-Wb14 and anti-WbT ELISA assays displayed similar sensitivity and specificity to the Og4C3 and POC-ICT tests and better specificity than the commercial antibody capture kit, BM14. Despite these promising results with the Brazilian sera, both will need to be tested with samples from other filarial and related parasitic worms, such as *O. volvulus*, *L. loa*, *B. malayi* and *B. timori*, so that in the future they may be considered a viable alternative to be used in other countries endemic for lymphatic filariasis.
